# An efficient lightweight network for image denoising using progressive residual and convolutional attention feature fusion

**DOI:** 10.1038/s41598-024-60139-x

**Published:** 2024-04-25

**Authors:** Wang Tiantian, Zhihua Hu, Yurong Guan

**Affiliations:** 1 School of Computer and Software Engineering, Sias University, Zhengzhou, 451150 Henan China; 2 School of Computer, Huanggang Normal University, Huanggang, 438000 Hubei China

**Keywords:** Image denoising, Deep learning, Image processing, Residual learning, Computer vision, Computer science, Information technology, Software

## Abstract

While deep learning has become the go-to method for image denoising due to its impressive noise removal capabilities, excessive network depth often plagues existing approaches, leading to significant computational burdens. To address this critical bottleneck, we propose a novel lightweight progressive residual and attention mechanism fusion network that effectively alleviates these limitations. This architecture tackles both Gaussian and real-world image noise with exceptional efficacy. Initiated through dense blocks (DB) tasked with discerning the noise distribution, this approach substantially reduces network parameters while comprehensively extracting local image features. The network then adopts a progressive strategy, whereby shallow convolutional features are incrementally integrated with deeper features, establishing a residual fusion framework adept at extracting encompassing global features relevant to noise characteristics. The process concludes by integrating the output feature maps from each DB and the robust edge features from the convolutional attention feature fusion module (CAFFM). These combined elements are then directed to the reconstruction layer, ultimately producing the final denoised image. Empirical analyses conducted in environments characterized by Gaussian white noise and natural noise, spanning noise levels 15–50, indicate a marked enhancement in performance. This assertion is quantitatively corroborated by increased average values in metrics such as Peak Signal-to-Noise Ratio (PSNR), Structural Similarity Index (SSIM), and Feature Similarity Index for Color images (FSIMc), outperforming the outcomes of more than 20 existing methods across six varied datasets. Collectively, the network delineated in this research exhibits exceptional adeptness in image denoising. Simultaneously, it adeptly preserves essential image features such as edges and textures, thereby signifying a notable progression in the domain of image processing. The proposed model finds applicability in a range of image-centric domains, encompassing image processing, computer vision, video analysis, and pattern recognition.

## Introduction

Image denoising involves the removal of unwanted noise from images, a crucial process in applications ranging from surveillance and transportation to medical care. The introduction of noise during image acquisition is inevitable, resulting from limitations in the imaging environment and the equipment used. Given these constraints, noise removal becomes an imperative step, whether the aim is to achieve visually appealing images or to prepare images for subsequent computer vision tasks such as image segmentation, recognition, and target detection^1^.

Historically, image denoising has been a classic inverse problem within the realm of computer vision^2^. Over the years, a plethora of effective methods have been proposed to address this challenge. These methods can be broadly categorized into two types: model-based and learning-based approaches^3^. Model-based methods involve modeling the distribution of natural images or the noise itself. Once modeled, this distribution is used as the prior, and optimization algorithms are then employed to generate clearer images. Frequently used prior features in this domain include local smoothness, sparsity, non-local self-similarity, and external statistical priors. In particular, non-local self-similarity and sparsity have been instrumental in enhancing the performance of image denoising methods. For instance, the Non-Local Means (NLM) technique^4^ identifies and averages similar regions within an image to effectively mitigate Gaussian noise. Similarly, the Block-Matching and 3D Filtering (BM3D) approach^5,6^ identifies similar two-dimensional image blocks and then processes these blocks in three-dimensional groups to produce a denoised image. The Weighted Nuclear Norm Minimization (WNNM) method^7^ stands out for its ability to preserve intricate texture details while significantly reducing noise. However, while these methods have shown promise, they come with their own set of challenges. These include the necessity for manual parameter tuning and the reliance on computationally expensive optimization algorithms.

Deep learning emerges as a pivotal solution in this context. Owing to their intrinsic flexibility and powerful learning capabilities, deep neural network architectures stand out as optimal solutions for the challenges previously highlighted in image denoising. The advent and progressive development of deep neural networks have catalyzed substantial advancements in learning-based denoising methods^8–11^, marking a significant evolution in this field. For instance, the Denoising Convolutional Neural Network (DnCNN)^12^ incorporates residual learning (RL) and batch normalization, enabling faster convergence and superior performance. However, increasing the depth of such networks can sometimes lead to diminishing returns in terms of performance. To counter this, techniques like the Deep Recursive Network(DRN)^13^ and the Fast and Flexible Denoising Network (FFDNet)^14^ have been introduced. Another notable method, the Convolutional Blind Denoising (CBDNet)^15^, offers a holistic approach, factoring in both synthetic and real noise during network training, thereby elevating the denoising efficacy and generalizability of the network.

Despite these advancements, challenges in image denoising persist:

- Achieving a balance between preserving spatial details and maintaining high-level context remains elusive. Many denoising networks rely on single-scale local convolutions, leading to a limited receptive field and potentially inconsistent semantic outputs.
- Edge preservation is a concern, with many techniques resulting in the unintended smoothing of edges and loss of critical edge information.
- There is a missed opportunity in leveraging the rich feature information from shallow models within deeper networks, leading to suboptimal denoising outcomes.

To address these challenges, this study tackles the prevalent challenge of high computational complexity caused by the excessive depth of networks in current deep learning-based denoising approaches. By integrating the strengths of DB and RL^16^, along with a progressive fusion tactic, we introduced a residual fusion dense network specifically designed for the elimination of Gaussian and real-world noise. Diverging from the well known DnCNN^12^ approach that primarily relies on a straightforward concatenation of convolutional layers for noise reduction, our method strategically implements densely interconnected DB within the network. Each layer of this network is engineered to process the feature maps from preceding layers, utilizing a progressive methodology to systematically link shallow convolutional features with deeper features extracted from each DB, thereby creating residual blocks. Within the CAFFM, a tripartite attention mechanism generates relative attention weights that capture the interrelations among three dimensions. These weights are subsequently applied and distributed to the pair of feature planes designated for fusion. This non-linear approach to feature fusion discerns the interplay among various feature planes, thereby significantly enhancing the efficacy of the fusion process. This design significantly enhances the network’s ability to accurately predict noise distribution. Moreover, the densely connected structure substantially lowers the computational complexity, reduces the overall number of network parameters, and effectively shortens the algorithm’s computation time.

In short, the key contributions of the proposed model are as follows:

- By combining DBs with RL,Our approach utilizes dense connectivity for enhanced feature extraction and residual connections to maintain information flow, allowing the network to learn more effective denoising functions at greater depths without the usual performance decline.
- Our model introduces a unique progressive residual fusion strategy that combines surface-level and deeply-extracted features, ensuring thorough use of information and enhancing its ability to robustly denoise a broad spectrum of noise types and intensities.
- The integration of CAFFM precisely captures and merges features across dimensions, overcoming existing attention mechanisms’ limitations by analyzing the relationships between channel, height, and width dimensions. This allows for a refined adaptation to the dynamic aspects of image features and noise.
- Through the strategic deployment of bottleneck structures and weighted averaging within CAFFM, our model not only reduces computational load but also significantly improves the quality of feature fusion. This leads to a more efficient network that does not compromise on denoising performance.

## Related research

In recent years, significant advancements have been made in the field of image denoising, leading to the emergence of a plethora of sophisticated algorithms. Broadly, these algorithms can be categorized into two primary groups: the conventional methods reliant on artificial features, and those anchored in deep learning techniques^17^. Moreover, within the realm of traditional image denoising, methods have been largely rooted in artificial features and can be categorized into two distinct approaches: the spatial domain and the transform domain.

**1. Spatial Domain Denoising:** One technique that has gained prominence in this category is the neighborhood mean method. At its heart, this method revolves around the principle of leveraging the average values within a neighborhood to execute approximate calculations. This approach is tailored to combat and eliminate noise that manifests itself through local similarities in images. While the method is proficient in neutralizing certain types of localized and random similarities in noise, it occasionally falters by overlooking intricate, localized details that may be inherent in the image.

**2. Transform Domain Denoising:** Transitioning to the transform domain, the methodology is predicated on the ability to represent genuine image signals with a minimal set of linear elements. By invoking specific transformations, notably the discrete cosine transform and wavelet transform^18^, this technique transposes genuine image signals into the transform domain. An insightful advancement in this domain was presented by Luo et al.^19^. They brought to the fore a hybrid adaptive image denoising technique. Central to this approach is the proactive learning from prior images. This strategy not only offers the potential for reduced computational complexity through algorithmic simplification but also poses challenges. Specifically, the identification of suitable priors becomes cumbersome in scenarios populated by multiple images.

An overarching evaluation of these traditional methods illuminates certain challenges that cannot be overlooked. Notably, there’s a pronounced disparity between the encoded feature information they generate and the genuine characteristics of images. This divergence, compounded with their inherent rigidity, makes their adaptability in practical scenarios quite limited. Furthermore, the traditional extraction processes for image features are marred by their intricate nature and demand for excessive time and computational resources. With the multifaceted and intricate noise distributions observed in practical applications, these traditional techniques often find themselves ill-equipped to handle such challenges effectively^20^.

**(2) Deep Learning-Based Image Denoising Methods:** In recent advancements, a significant emphasis has been placed on image denoising techniques rooted in deep learning. These techniques are characterized by their formidable learning capabilities. They can adeptly accommodate noise of a more intricate distribution, offering the dual advantage of enhanced accuracy and reduced computational time, often surpassing the performance of traditional methods^21^.

One notable example is the model introduced by Kim et al.^22^. It leverages residual networks, which build upon earlier layers to progressively refine the image, and incorporates a convolutional attention module^23^. This module focuses on important image features, enhancing the network’s ability to distinguish noise from true details. While deep learning training improves denoising with this method, it can face challenges. Overdependence on residual connections can lead to overfitting, where the model memorizes training data instead of generalizing to unseen images

Pushing the boundaries further, Chen et al.^24^ presented a GAN-based model called GCBD. Its unique feature is the generator’s ability to create artificial noise blocks. These blocks are then combined with real images, significantly expanding the training dataset. This cleverly addresses the common issue of limited paired data (noisy and clean image pairs) available in real-world scenarios. Another GAN-based approach, ADGAN^25^ employed a feature loss function. This ensures that the denoised image retains the essence of the original, especially delicate details, by comparing specific features between the two. These examples showcase the diverse strategies employed in modern image denoising, each with its own strengths and limitations. Continued research in this area promises even more effective and robust methods for restoring pristine images from noisy data.

However, despite their demonstrated efficacy, a common characteristic shared by these denoising methods is their dependence on paired training datasets. This reliance presents a substantial obstacle, as obtaining such datasets proves challenging in practical applications. In response to this challenge, Li et al.^26^ utilized cycle consistent adversarial networks (CycleGAN)^27^ to denoise low-dose CT images without the requirement for paired training datasets. This innovative approach involved leveraging previously acquired full-dose CT images and aligning them with subsequent low-dose CT images from diverse patients, thereby enhancing denoising efficacy. However, there remains a need for further refinement of this methodology, particularly in preserving image details such as edges, textures, and ensuring overall image fidelity.

Within the domain of CycleGAN, CycleWGAN^28^ has emerged as a supervised learning variant. By substituting JS divergence and period loss in the original CycleGAN network with Wasserstein distance and introducing a supervised loss, it effectively addresses the issue of mode collapse. Nevertheless, this method does require prolonged training durations. Another derivative, CaGAN^29^, employed dual attention modules to reinforce feature correlation in spatial and channel dimensions, concurrently refining the loss function. Simultaneously, in their quest to enhance training stability, Li et al.^30^ replaced the adversarial loss in the original CycleGAN with a least squares loss function. Their approach decentralizes image discrimination by evaluating individual patches before amalgamating their outcomes for a comprehensive result. This not only streamlines the discriminator structure but also facilitates superior learning of image details.

Finally, Tan et al.^31^ proposed an unsupervised denoising paradigm based on CycleGAN, enriched with a bilateral network in selective kernel networks (SK-NET) to selectively choose features. By incorporating a patchGAN discriminator and perceptual loss, this model ensures that the processed images closely mirror the intricate details of the original images.

## Background

In the subsequent subsections, we delve into the intricacies of previous CNN modules that hold significant potential for integration into our proposed model. Understanding these modules is essential to appreciate the novelty and robustness of our approach. We encourage readers to pay close attention to these foundational elements.

### Motivation

The evolution of CNNs has significantly propelled the field of image denoising, offering advanced solutions that surpass traditional methods. Traditional approaches often struggle to extract intricate image features or adapt to the diverse nature of noise, leading to suboptimal denoising results. Recognizing these challenges, the motivation for our proposed method emerges from the desire to enhance feature extraction capabilities and computational efficiency in the denoising process. DB and RL principles have shown promise in addressing these issues, yet their full potential remains untapped in the context of image denoising. The introduction of DB offers a way to leverage feature richness through layer interconnectivity, while RL promises to counteract the degradation of network performance with depth. However, the integration of these modules lacks a cohesive framework that can efficiently and effectively utilize both local and global features for superior denoising performance. This gap underscores the need for a novel approach that can harness the strengths of both DB and RL, along with advanced attention mechanisms, to set a new benchmark in image denoising.

### Dense blocks

**Figure 1:**
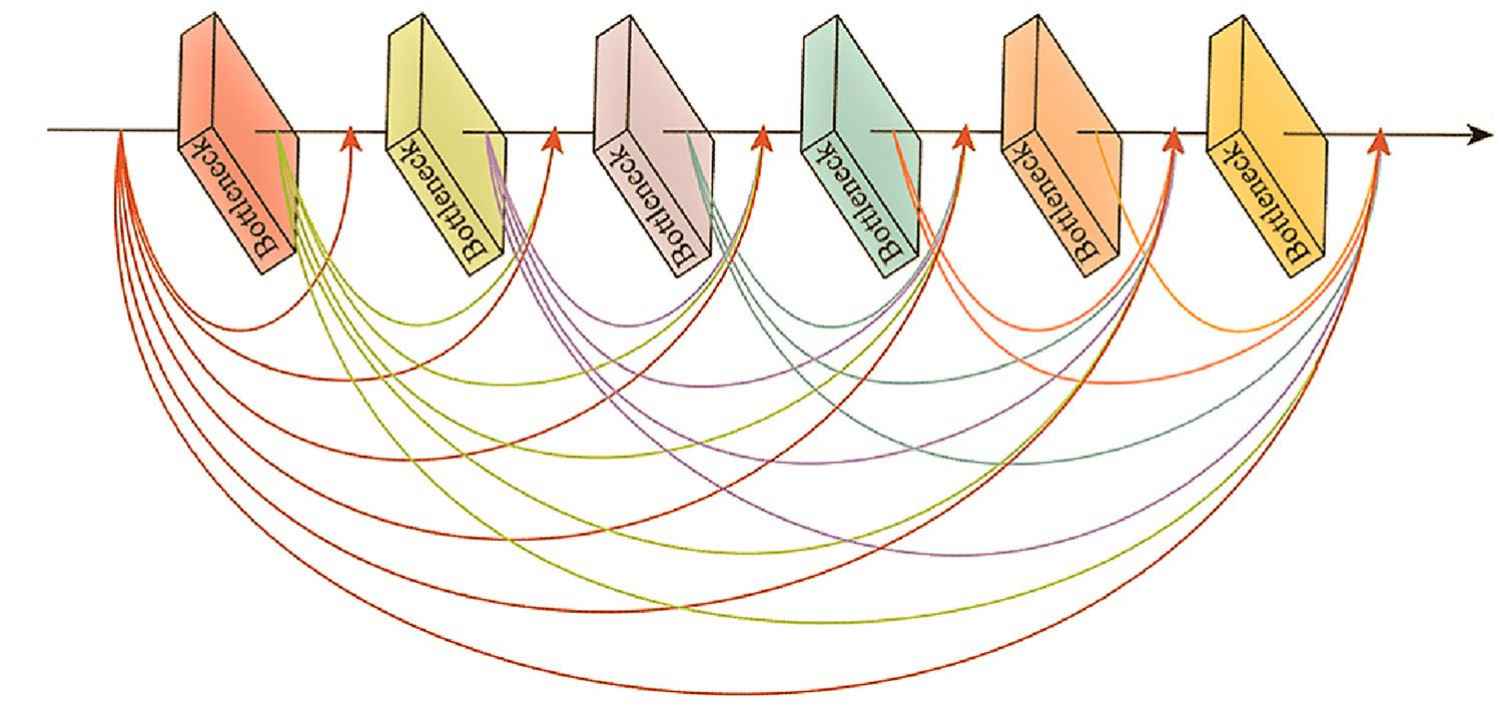
Graphical representation of dense block.

**Figure 2:**
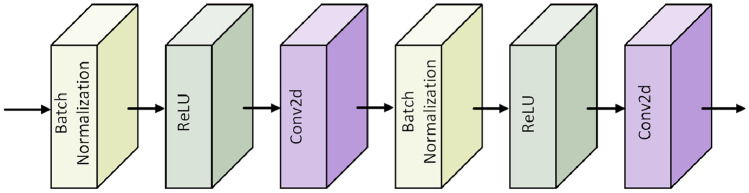
Overall structure of bottleneck.

CNNs consist of three components: input layer, hidden layer, and output layer. The main structure of the hidden layer alternates between linear convolution and non-linear activation functions, primarily serving to map features from the input. In the domain of image denoising, the advantage of CNNs over other traditional methods is that the hidden layer can better extract image features. The shared weights significantly reduce the computational burden of the network model, effectively reducing the number of network parameters, resulting in a more efficient model. Taking into consideration how to extract more image features while also significantly reducing the computation parameters of the model, a dense network was designed. The core module in this network is the DB^32^. Its structure is shown in Fig. 1, and the structure of the Bottleneck module is illustrated in Fig. 2. Within the DB structure, each layer is connected via short connections. The input for each layer comes from the output of all previous layers, and this connection can be represented by:

1 $$\begin{aligned} X_l = H_l([X_0,X_1,...,X_{l-1}]) \end{aligned}$$

where $X_l$ denotes the output feature map of the $l^{th}$ layer, and $[X_0,X_1,...,X_{l-1}]$ represents the channel-wise concatenation of the output feature maps from layer 0 to $(l-1)$, without any further operation on the channels. $(H_l)$ is a function that inputs the concatenated feature map into the BN. As the input of each layer accumulates outputs from all preceding layers, integrating all previously extracted feature maps, the input channels for subsequent layers will be relatively large. To reduce the number of input feature maps, a 1$\times$1 convolutional layer is designed within the BN. This not only minimizes parameters, reducing network computational cost, but also effectively merges features across channels, ensuring more efficient gradient propagation and comprehensive learning of noise distribution.

### Residual learning

The primary motivation behind RL is to address the problem of performance degradation in CNN as their depth increased. By learning residual mappings, residual networks ensure that training accuracy does not degrade with increased network depth, addressing the problem of network degradation. Considering the image denoising domain, the residual network is designed to learn noise images with relatively low information content through a skip-connection architecture. The noisy image is then subtracted from the predicted noise image by the network to obtain the underlying clean image, expressed by the equation:

2 $$\begin{aligned} y = x + n \end{aligned}$$

where *y* denotes the noisy image, *x* is the original clean image, and *n* represents additive noise. In the early stages of convolutional feature extraction, the design of the DB extracts rich image features, laying a foundation for subsequent learning of noise distribution.

## Proposed methodology

Inspired by the DenseNet network structure proposed by Huang et al.^33^, this paper uses dense networks to enrich the extracted image features and utilizes the dense short connection structure to reduce the computational complexity and the number of network parameters. After studying and replicating the denoising network based on residual DB proposed by Zhang et al.^34^, it was observed that the network did not fully utilize shallow convolutional features. Therefore, by adopting a progressive approach, three residual blocks are designed to merge shallow convolutional features with deep dense network-extracted features, ensuring that the deep dense network fully utilizes shallow features to learn noise distribution. Moreover, research into dense networks found that its structure did not integrate global features for learning. Inspired by the RDN network structure proposed by Zhang et al.^35^, a concatenation layer is designed before the reconstruction output layer. In this layer, the features that have been extracted by preceding dense networks are consolidated and then inputted into an attention mechanism. This mechanism guarantees that the network comprehensively assimilates both local and global features, which results in enhanced denoising outcomes. Further elaboration on the modules utilized in the proposed approach is provided in the subsequent sections.

### Network structure

**Figure 3:**
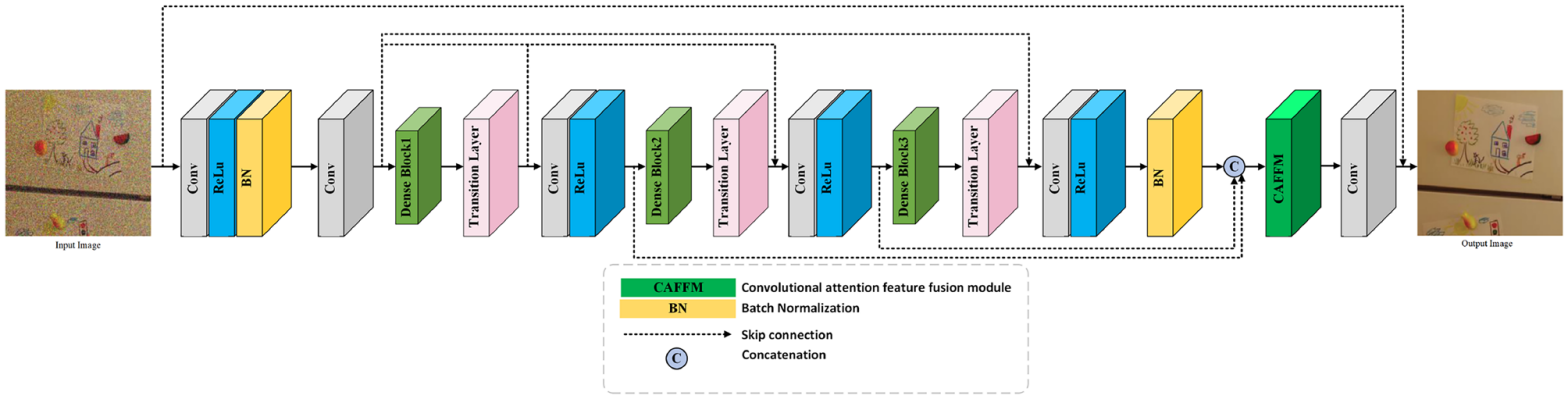
Overall structure of the proposed model.

This study provides insight into the progressive residual fusion dense network’s comprehensive architecture. The network comprises three DB modules that mirror each other structurally. Within each module, convolutional layers are followed by rectified linear unit (ReLU) activations, configured to address issues like vanishing gradients while reducing parameter interdependencies and instilling sparsity. An illustrative depiction of the network, shown in Fig. 3, details its design. Commencing with an initial convolutional layer employing a 3$\times$3 kernel with 64 filters paired with ReLU activation, this stratum extracts shallow image features. The subsequent layer maintains the kernel size but reduces filters to 24, regulating feature map channels and preempting excessive proliferation that could escalate computational demands. The network’s core comprises three DB modules, residual blocks, and interleaved Transition and ReLU+Conv layers across layers three through twelve. This multilayered structure facilitates intricate feature learning. Within DB, BN layers utilize 1$\times$1 and 3$\times$3 kernels with 48 and 12 filters respectively, allowing each layer to adaptively discern the noise distribution through fused features. The consistent 1$\times$1 kernel size in Transition Layers, equipped with 24 filters, refines parameter efficiency by consolidating channel features. The thirteenth layer, a Concatenation stratum, synergizes output maps from the first two DB with the final module to fortify global feature assimilation. Culminating in the fourteenth reconstruction output layer with a solitary 3$\times$3 kernel, this terminal stratum amalgamates forged global features while maintaining input dimensional congruence. This consistency underpins RL, empowering precise noise extraction and production of the denoised output image. The network’s meticulous design achieves a balance between feature extraction precision and computational efficiency, representing an advance in the domain.

### Attention mechanism

In recent years, the attention mechanism has become one of the hottest research directions in computer vision. The most representative is the SENet^36^, which captures the relationships between channels through the channel attention mechanism but neglects the important role of spatial attention information in feature representation. Subsequently, researchers have improved SENet by integrating attention information of different scales. Woo et al.^23^ proposed the Convolutional Block Attention Module (CBAM), which merges channel attention information with spatial attention information to create more robust feature attention representations. Dai et al.^37^ introduced Attentional Feature Fusion (AFF), which combines global and local channel attention information to adapt to features of different scales in images. However, these methods still did not consider the relationship between channel attention information and spatial attention information. For this reason, Hou et al.^38^ proposed the Stripe Pooling Network, which obtains the relationships between channels and width, and channels and height through stripe pooling layers. Misra et al.^39^ proposed the Convolutional Triplet Attention Module, learning the relationship between the three dimensions through a three-branch attention mechanism. Moreover, the attention mechanism also shows great potential in feature fusion. Liu et al.^40^ introduced Feature Pyramid Encoding Network that fuses deep channel attention information with shallow spatial attention information, merging semantic features and spatial details. AFF^37^ is used for feature fusion in both short and long skip connections. This module learns the relative attention weights between feature planes of different scales through the attention mechanism and merges them through nonlinear weighted fusion, significantly improving the network’s segmentation accuracy.

**Figure 4:**
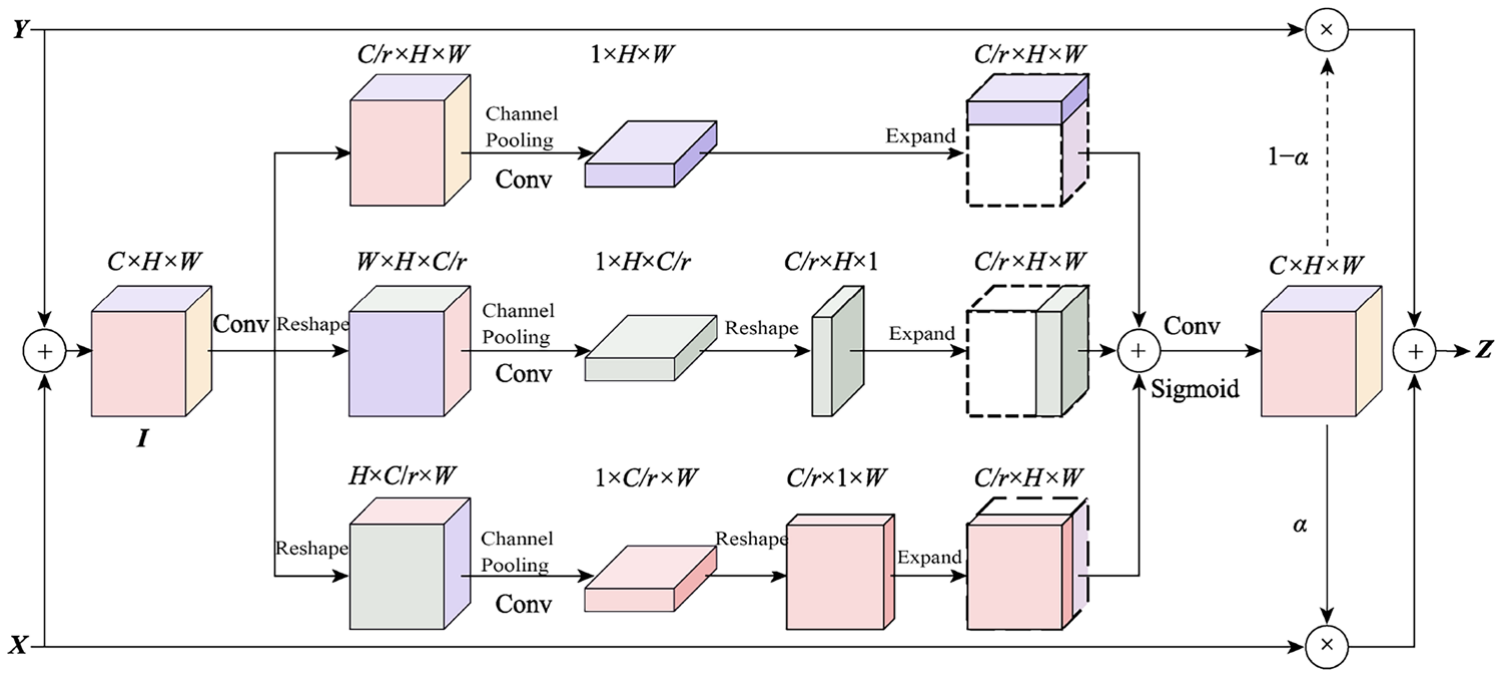
Graphical representation of convolutional attention feature fusion module.

Inspired by the prior model^39^, this paper proposed CAFFM, as shown in Fig. 4. While ensuring a small computational load, it improves the quality of feature fusion in lightweight convolutional neural networks. CAFFM captures the pairwise relationships between the three dimensions of channel, height, and width through a three-branch structure, generating three two-dimensional attention sub-maps, and merges them into one complete three-dimensional attention map to adapt to changes in feature information of different dimensions; finally, it merges feature planes of different scales through weighted averaging. In addition, it uses two 1$\times$1 convolutional layers to form a bottleneck structure, reducing the number of channels in the feature planes, further decreasing the computational load of CAFFM.

In CAFFM, given two feature planes $X, Y \in {\mathbb {R}}^{C \times H \times W}$, by default, assume *X* is the output feature plane from the shallow stage of the encoder and *Y* is from the deep stage. By element-wise addition, *X* and *Y* are first merged into an input tensor $I \in {\mathbb {R}}^{C \times H \times W}$, then *I* is processed by a $1 \times 1$ convolutional layer $P_{1}$ to obtain $T \in {\mathbb {R}}^{C/r \times H \times W}$, where *r* is the reduction ratio, the formula is:

3 $$\begin{aligned} T = \beta (P_1(I)) \end{aligned}$$

where $\beta$ represents BN; *r* is the reduction ratio mapping the tensor to a lower channel dimension space. *T* is fed into three separate branches, each capturing the pairwise relationships between channels, height, and width, and finally merging into a three-dimensional tensor.

The first branch learns the relationship between the height and width dimensions. By encoding *T* in the channel dimension through channel average pooling, we obtain $T_{h,w} \in {\mathbb {R}}^{1 \times H \times W}$, specifically,

4 $$\begin{aligned} T_{h,w} = \frac{1}{C} \sum _{0 \le i < C} T_{i,j,k} \end{aligned}$$

where $T_{h,w}$ is then processed by a 7$\times$7 standard convolutional layer $S_1$, obtaining a two-dimensional tensor $O_{h,w} \in {\mathbb {R}}^{1 \times H \times W}$ that contains the relationship between the two dimensions of height and width, i.e.,

5 $$\begin{aligned} O_{h,w} = \beta (S_1(O_{h,w})) \end{aligned}$$

The second branch learns the relationship between the channel and height dimensions. Similar to the first branch, but encoding *T* in the width dimension and reshaping it to ${\mathbb {R}}^{W \times H \times C/r}$, then through channel average pooling, we obtain $T_{c,w}$, and after passing it through a 7x7 standard convolutional layer, we get $O_{c,w} \in {\mathbb {R}}^{1 \times C/r \times W}$. After reshaping, we obtain $O_{c,w} \in {\mathbb {R}}^{C/r \times H \times 1}$, and finally, we extend it to $O_2 \in {\mathbb {R}}^{C/r \times H \times W}$. The third branch learns the relationship between the channel and width dimensions. By reshaping *T* into ${\mathbb {R}}^{H \times C/r \times W}$ and encoding it through channel average pooling on the height dimension, we obtain $T_{c,w}$. After processing through a 7$\times$7 convolutional layer, we get $O_{c,w}$, and after reshaping and extending, we obtain $O_3 \in {\mathbb {R}}^{C/r \times H \times W}$. By element-wise addition and arithmetic averaging, we merge the output tensors of the three branches, which have the same shape, into a three-dimensional tensor $O \in {\mathbb {R}}^{C/r \times H \times W}$, containing the complete relationships between the dimensions, i.e.,

6 $$\begin{aligned} O = \frac{1}{3}(O_1 \oplus O_2 \oplus O_3) \end{aligned}$$

where $\oplus$ represents element-wise addition. To integrate the global context information of the three output tensors and restore the channel number of *O* to the same as *X* and *Y*, we introduce a 1$\times$1 convolutional layer $P_2$; then through a Sigmoid activation function $\sigma$, we obtain a three-dimensional attention map $\alpha \in {\mathbb {R}}^{C \times H \times W}$, i.e.,

7 $$\begin{aligned} \alpha = \sigma (\beta (P_2(O))) \end{aligned}$$

The attention map is then weighted and assigned to *X* and *Y*, obtaining the output tensor $Z \in {\mathbb {R}}^{C \times H \times W}$,

8 $$\begin{aligned} Z = \alpha \odot X + (1 - \alpha ) \odot Y \end{aligned}$$

where $\odot$ represents element-wise multiplication; the weights in $\alpha$ and $1 - \alpha$ range from 0 to 1, and after being assigned to *X* and *Y*, the sum of each position is 1, which can be considered as a weighted average between *X* and *Y*.

### Image denoising algorithm based on residual fusion

**Figure 5:**
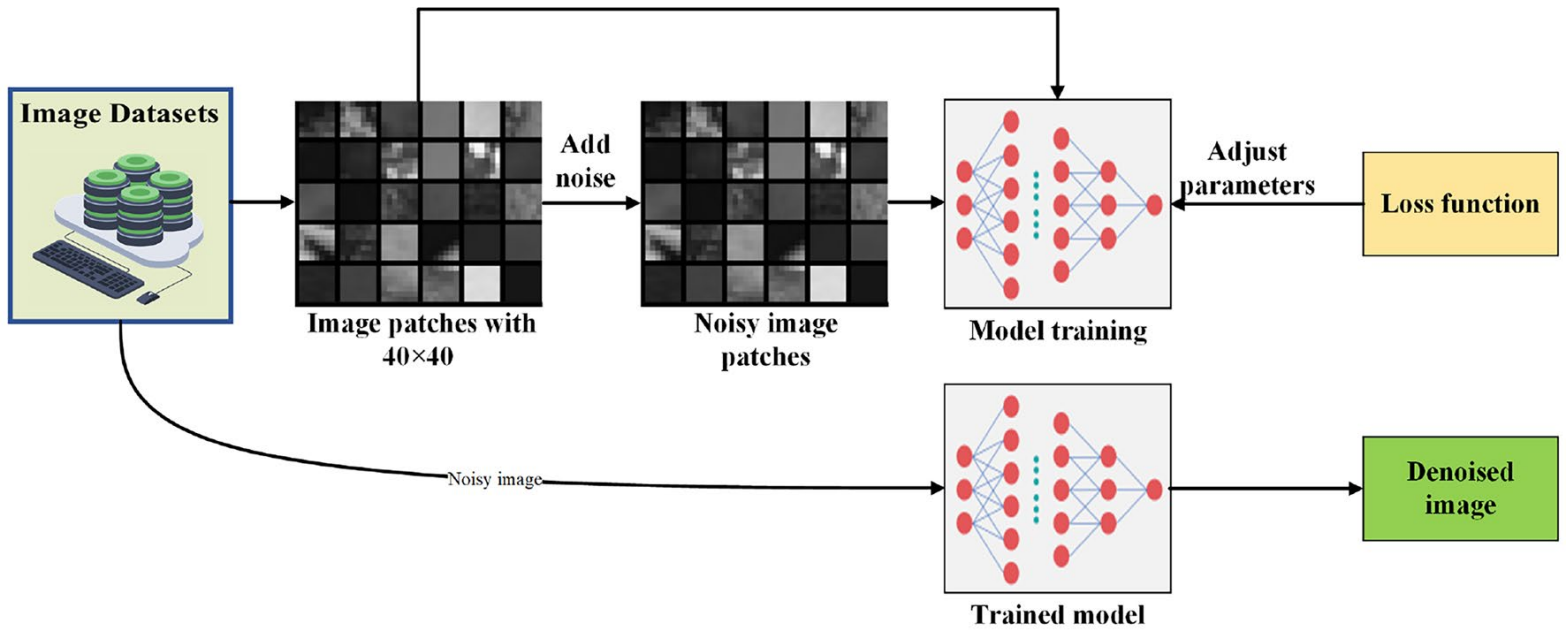
Structure of the image denoising technique utilizing residual fusion.

The specific flow of the algorithm is shown in Fig. 5. During the training process, the original image is cropped into image blocks of the same size. These original image blocks, added with noise, are input into the designed network. Through the loss function, parameters are adjusted via backpropagation until the network converges. In the testing phase, noisy images are input into the already converged network to directly output the corresponding predicted denoised images. The loss function expression used in this paper’s algorithm is:

9 $$\begin{aligned} L_{loss} = \frac{2}{N}\sum _{i=1}^{N} || R(y_i,\Theta )-(y_i - x_i) ||^2_F \end{aligned}$$

where $$R(y_i,\Theta )$$ is the estimated residual image of the noise input, $$ y_i$$ is the input noisy image, and $$x_i$$ is the clean image. $$(y_i-x_i)$$ gives the standard residual image. *N* represents the number of input samples in a batch. The training process continuously iterates to reduce the loss function, i.e., reduce the error between the estimated residual and the standard residual. In this way, the predicted denoised image will be closer to the original clean image, achieving better denoising effects.

## Different modules and their combinations

Our proposed methodology innovatively integrates DB, RL, and the CAFFM to set a new benchmark in image denoising. This section delves into the rationale behind this specific combination, highlighting how their synergistic interaction leads to unparalleled denoising performance.

### Complementarity of components

The core strength of our model lies in the complementary nature of its components. DB ensure that our network can extract a rich set of features from the input image by leveraging the outputs of all preceding layers, thus creating a comprehensive feature set. This is critical for understanding the complex nature of image noise and for extracting the underlying clean image information. RL, on the other hand, addresses the challenge of training deeper networks without succumbing to performance degradation. By learning the residual noise instead of the clean image directly, our model efficiently identifies and filters out noise, even in cases where it is subtly intertwined with image content. This approach not only simplifies the learning objective but also enhances the model’s ability to generalize across different noise patterns. The inclusion of CAFFM brings a focused efficiency to the model. By implementing an attention mechanism, CAFFM directs the model’s computational resources toward features most relevant for denoising, ensuring that the network does not get overwhelmed by irrelevant data. This results in a more precise denoising process, as the model learns to prioritize and refine features that significantly contribute to noise reduction.

### Synergistic effects

The synergy between DB, RL, and CAFFM is not merely additive but multiplicative in terms of enhancing model performance. DB provide a rich feature base that is essential for any denoising task. RL optimizes the network’s depth, ensuring that even the subtlest noise can be identified and removed. Similarly, CAFFM, through its attention mechanism, acts as a good filter for the better focus of the model to find important features contributing most importantly towards the process of denoising. This combination ensures that our model is not just deep but also smart in processing information. By efficiently managing computational resources and focusing on the most relevant features, our model achieves a high degree of precision in noise identification and removal, outperforming existing models that might rely on depth or feature richness alone.

### Empirical validation

**Figure 6:**
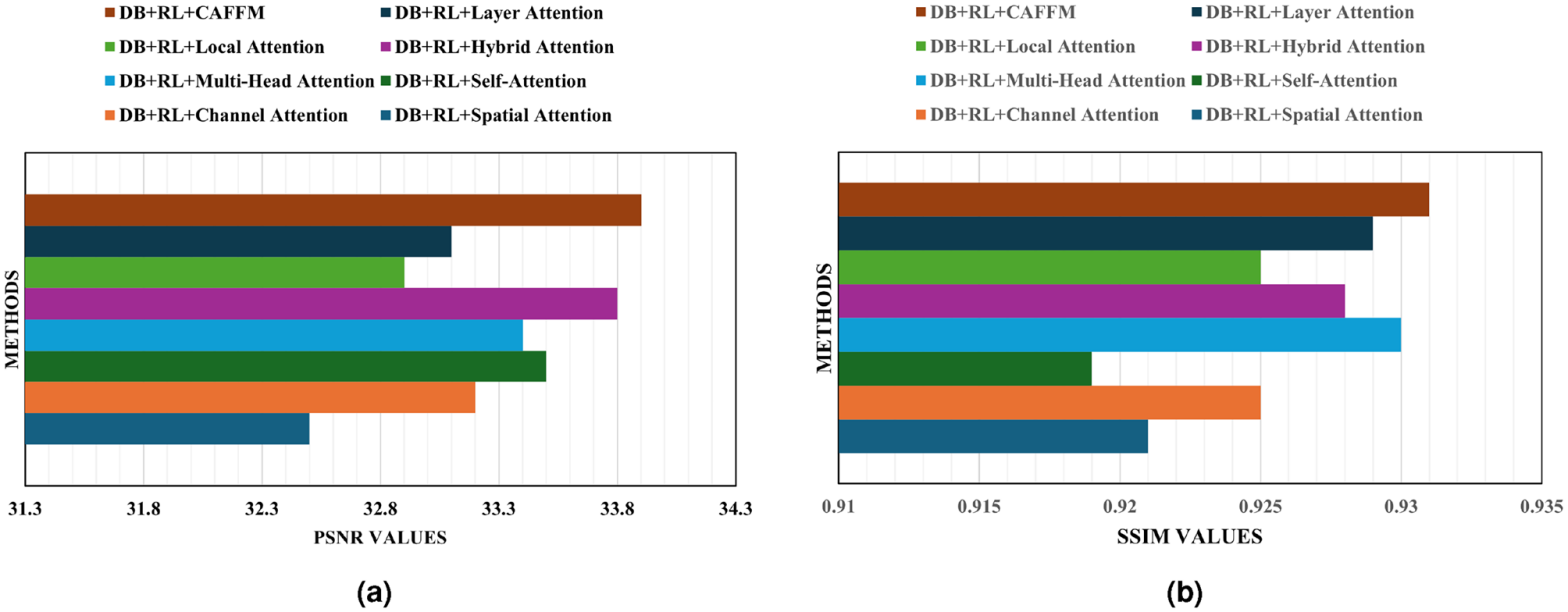
Quantitative analysis of PSNR and SSIM on Set12, BSD68, and Kodat24 datasets.

Empirical evidence underscores the success of combining DB, RL, and CAFFM within our model. This combination’s efficacy is further scrutinized by examining alternative attention mechanisms such as Spatial Attention, Channel Attention, Self-Attention, Multi-Head Attention, Hybrid Attention, Local Attention, and Layer Attention-paired with DB and RL. Through this comprehensive validation as shown in Fig. 6, it became clear that our selected integration of technologies outperformed others across various datasets. This deeper analysis not only confirmed our initial findings but also highlighted the unique effectiveness of our chosen blend in enhancing model performance.

In short, the integration of DB, RL, and CAFFM into our model represents a holistic approach to the challenge of image denoising. By leveraging the unique strengths and synergistic potential of these components, our model attained superior performance, setting a new standard in the field.

## Experimental results

### Implementation details

In this study, a total of 33,725 images are meticulously chosen for the training of the neural network. These images include contributions from both the ImageNet^41^ and BSD400^42^ datasets. Additionally, grayscale images affected by noise, as well as their colored counterparts, are included in the training set, each sized at 180$\times$180 pixels. These images cover a range of subjects like natural scenery, animals, people, and buildings. To improve the training efficiency and speed up convergence, we used a cropping technique. This involved setting the crop size to 40 and a stride of 10. This approach resulted in 380 images being cropped into 215,552 smaller images, each 40$\times$40 pixels, which form the main part of the training dataset. We also set aside 20 images for validation to test the network’s performance and utility with different test sets. The test set for the experiment is randomly selected from the Set12, Set68^12^, DND^43^ datasets.

Experiment parameters: batch size is 64, training 33,725 sample data per epoch, epoch is 150, learning rate is fixed at 0.001. The computer’s CPU is Intel Core i7, GPU is GTX1080Ti, RAM is 11 GB, OS is Windows 10, and the network is trained, verified, and tested on the PyTorch deep learning framework. This framework can use GPU acceleration to save training time. The software used for training and testing is PyCharm, and the Python version is 3.9.

### Measurement standards

The evaluation standards used in the experiment include subjective and objective evaluations. Subjective evaluation refers to visual inspection of images, evaluating the denoising effect of the model’s output image. Objective evaluation uses peak signal to noise ratio (PSNR) and structural similarity (SSIM). PSNR, based on mean square error (MSE), is an image quality evaluation index^2^. The higher the PSNR value, the better the image quality. In the experiment, the higher the PSNR value indicates a higher similarity between the denoised image and the original image. PSNR is calculated as:

10 $$\begin{aligned} MSE= & {} \frac{1}{H \times W} \sum _{j=1}^{H} \sum _{k=1}^{W} (M(j,k) - N(j,k))^2 \end{aligned}$$

11 $$\begin{aligned} PSNR= & {} 10 \log \left( \frac{2^n}{MSE} \right) ^2 \end{aligned}$$

where *M* and *N* are the predicted and true values, respectively. *j* and *k* are all pixels in the image. *H* and *W* represent the height and width of the image, and *n* is set to 8. Structural Similarity (SSIM) is an evaluation metric to measure the similarity between two images. It estimates similarity based on image brightness, contrast, and structure. The mean is used as the brightness estimate, the standard deviation as the contrast estimate, and the covariance as the measure of structural similarity. SSIM is calculated as:

12 $$\begin{aligned} SSIM(M,N) = \frac{(2\mu _m \mu _n +c1)(2\sigma _{mn} +c2)}{(\mu _m^2 +\mu _n^2 +c1)(\sigma _m^2 +\sigma _n^2 +c2)} \end{aligned}$$

where $\mu _m$ is the mean of image M, and $\mu _n$ is the mean of image N. $\sigma _m^2$ and $\sigma _n^2$ represent the variances of images M and N, respectively. $\sigma _{mn}$ represents the covariance between images *M* and *N*. *c*1 and *c*2 are constants for stability. The SSIM value ranges between 0 and 1, with a higher SSIM indicating more similarity. When SSIM is 1, the two images being compared are identical.

Furthermore, we also utilized Feature Similarity Index for Color images (FSIMc), is quantitatively evaluate the score of image quality. In simple words, it can be used to measure the similarity between two color images. The FSIMc is an extension of the FSIM, which is originally designed for grayscale images. The equation for FSIMc typically looks something like this:

13 $$\begin{aligned} FSIMc(X, Y) = \frac{\sum _{x=1}^{M} \sum _{y=1}^{N} PC_m(x,y) \cdot GM_m(x,y) \cdot max\{S_l(x,y), S_r(x,y)\}}{\sum _{x=1}^{M} \sum _{y=1}^{N} PC_m(x,y) \cdot GM_m(x,y)} \end{aligned}$$

where *X* and *Y* are the two color images being compared, $PC_m$ is the phase congruency at a given pixel, $GM_m$ is the gradient magnitude at the pixel, and $S_l$ and $S_r$ are the similarity measures for the left and right images respectively. The sums are taken over all pixels (*x*, *y*) in the images. The FSIMc score is a value between 0 and 1, where a higher value indicates greater similarity between the two images. This makes it suitable for assessing the effectiveness of algorithms in tasks like image compression, watermarking, and denoising, especially where color fidelity is crucial.

**Figure 7:**
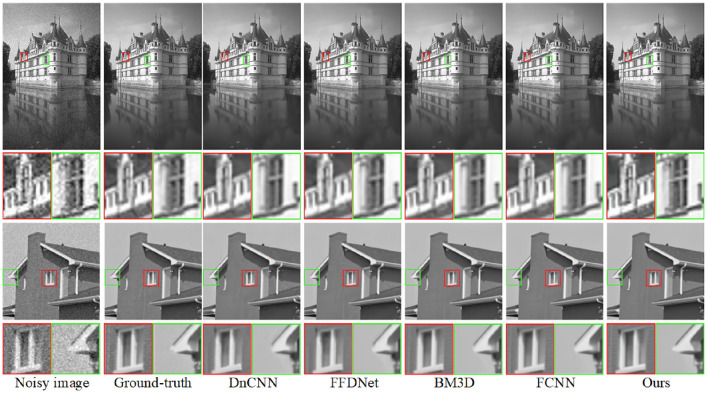
Visual analysis of grayscale image denoising using the Set12 and BSD68 datasets with noise level $\sigma =25, 50$.

### Qualitative and quantitative results

To compare traditional denoising techniques with deep neural network-based methods, we conducted both quantitative and qualitative evaluations on diverse datasets. The quantitative assessment involved using important metrics such as PSNR, SSIM and FSIMc to numerically evaluate the quality of the denoised images. Additionally, we performed a qualitative evaluation by visually representing the restored images, allowing for an intuitive understanding of their visual quality and accuracy. This comprehensive evaluation approach provides valuable insights into the denoising capabilities of different techniques across various datasets, making a significant contribution to image processing research.

The objective of this study is to systematically assess the denoising performance of the proposed algorithm. This dual-level assessment allows for a thorough understanding of each algorithm’s performance under varying noise intensities. Using both subjective and objective measures, the study aims to provide a holistic view of the denoising capabilities.

**Figure 8:**
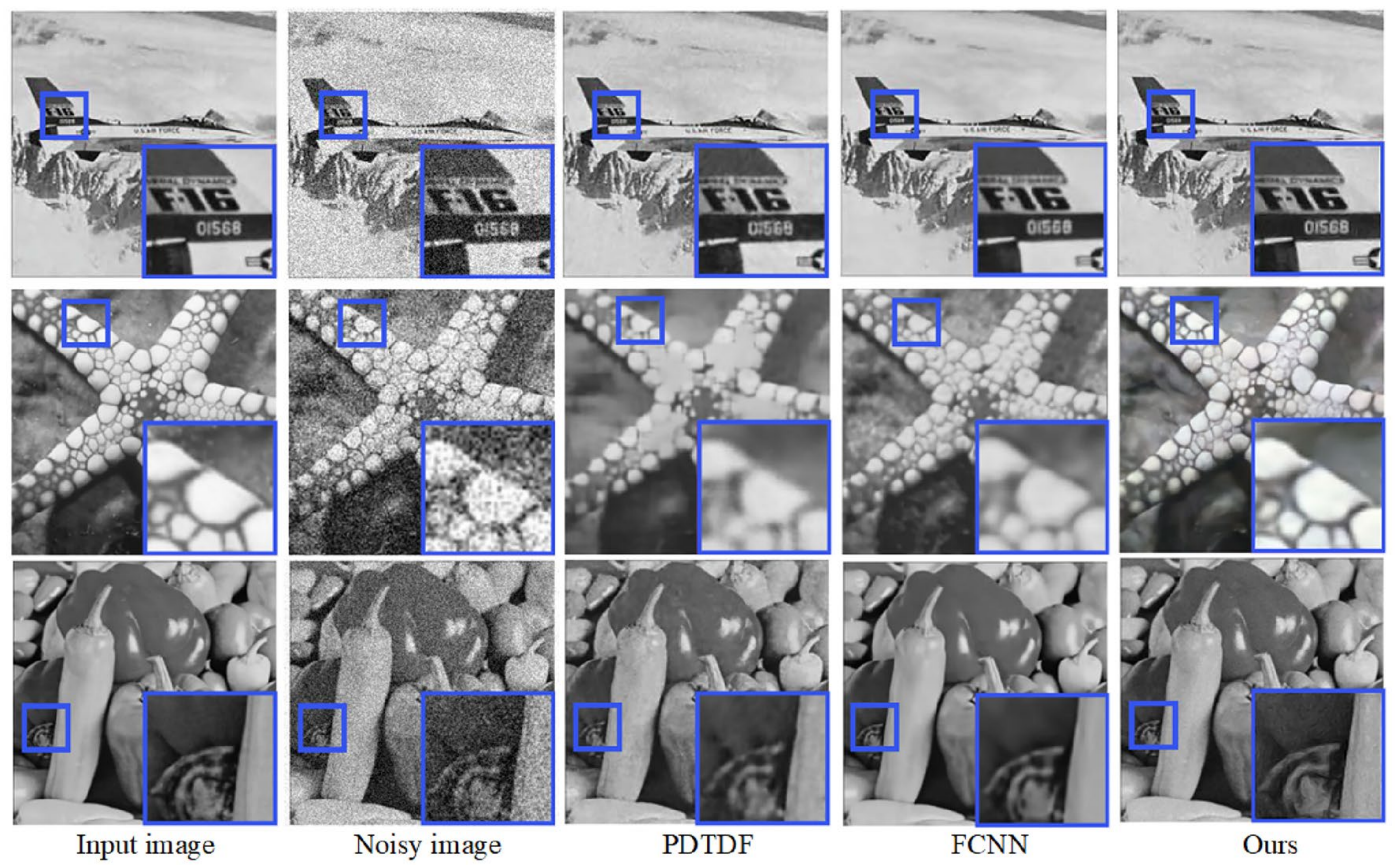
Visual outcomes of grayscale images using Set12 dataset with noise level $\sigma =25$.

**Figure 9:**
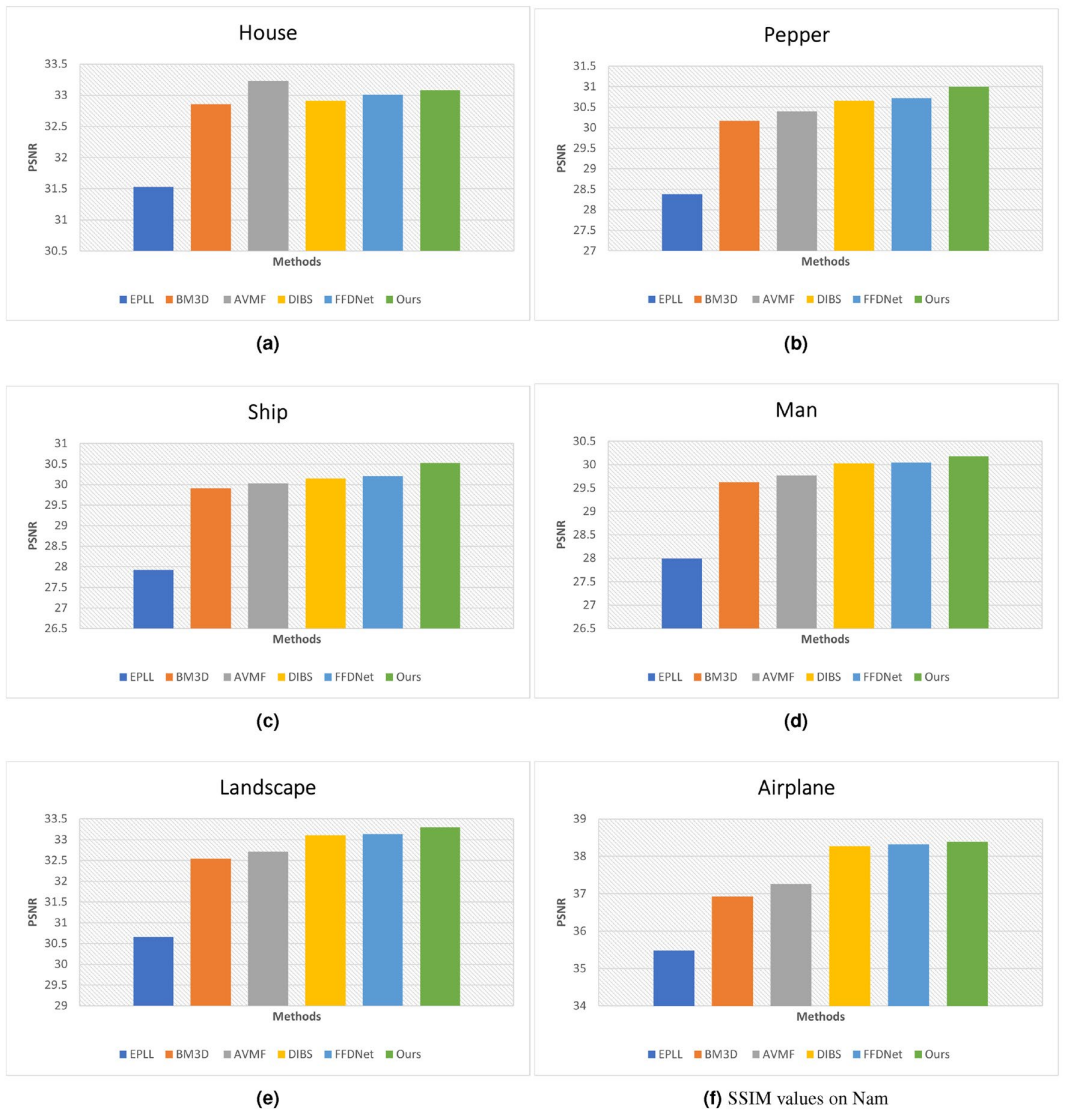
Quantitative analysis of PSNR on the Set12 dataset with noise level $\sigma =25$. (**a**) House. (**b**) Pepper. (**c**) Ship. (**d**) Man. (**e**) Landscape. (**f**) Airplane.

**Figure 10:**
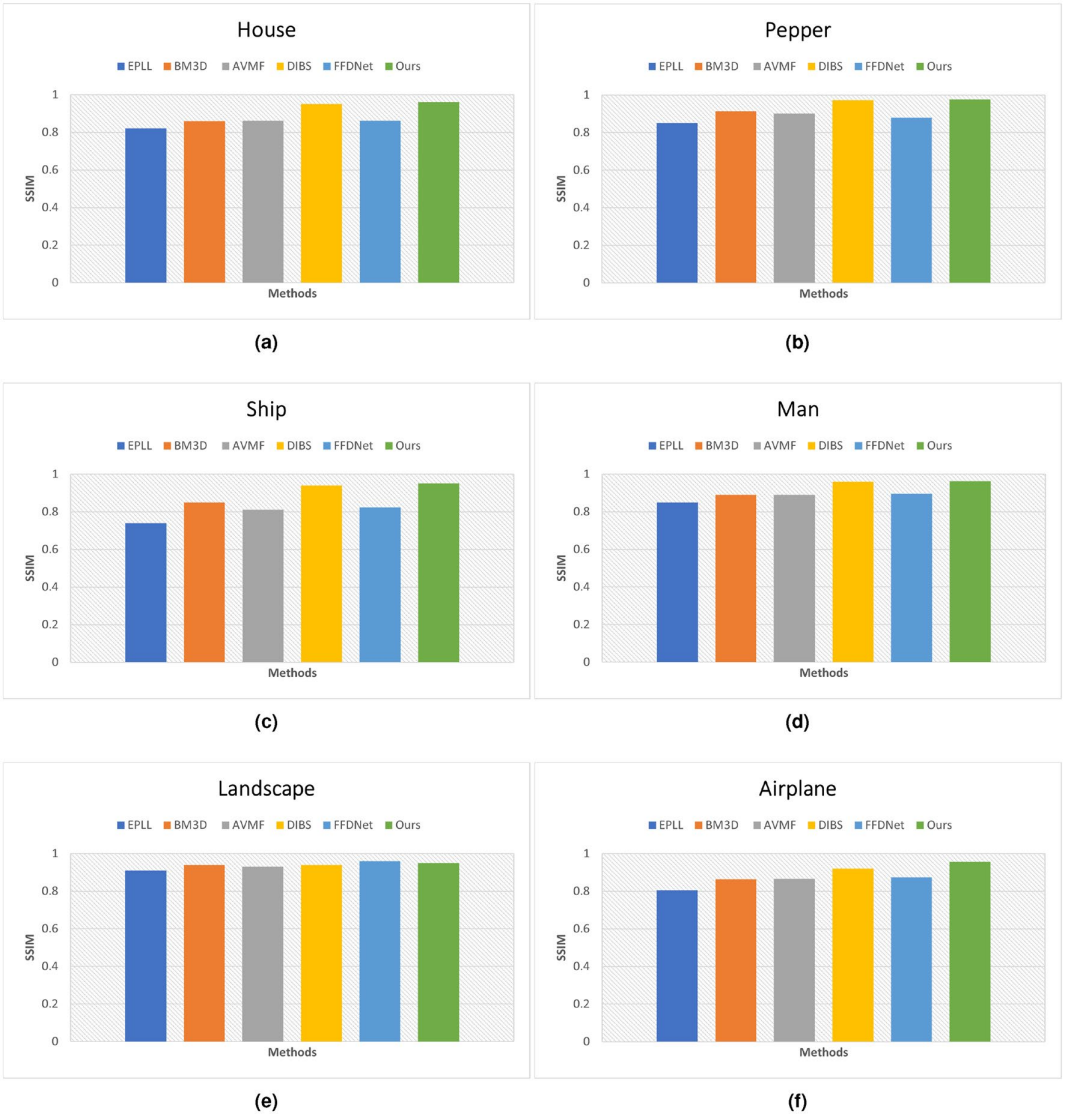
Quantitative analysis of SSIM on the Set12 dataset with noise level $\sigma =25$. (**a**) House. (**b**) Pepper. (**c**) Ship. (**d**) Man. (**e**) Landscape. (**f**) Airplane.

#### Grayscale image denoising

Initially, the efficacy of both the proposed and prior approaches is evaluated through experiments conducted on grayscale images taken from the Set12 and BSD68 datasets. The visual results from these models are illustrated in Fig. 7. We expanded our analysis by comparing our method with established techniques such as DnCNN^12^, FFDNet^14^, BM3D^5^, and FCNN^44^. The denoising efficacy of each mentioned algorithms is evaluated under conditions of Gaussian white noise at levels of $\sigma = 25$ and $\sigma = 50$. In the analysis of the region of interest (ROI) depicted in Fig. 7, marked by red and green rectangles, it becomes clear that algorithms such as DnCNN, FFDNet, and BM3D tend to oversmooth the image’s edges, resulting in diminished clarity of the content. In contrast, the FCNN’s visual outputs exhibit enhanced texture and structural definition. Building upon this comparative observation, our proposed method exhibits a notably superior performance. It distinctly excels in preserving sharpness along the edges and capturing intricate details. Simultaneously, it upholds visual fidelity, especially in the smoother areas of the images, thus striking a balance between detail preservation and smoothness.

Moreover, the efficacy of our proposed method is further validated through a comparative analysis with two models, namely PDTDF^45^ and FCNN^44^. The images utilized for this experiment are sourced from Set12 and Set14 datasets. A thorough examination of the visual outcomes in Fig. 8 revealed that while PDTDF and FCNN succeed in restoring images to a significant degree, they tend to oversmooth edges and textures. This observation leads to a nuanced understanding of their limitations in preserving fine details. In contrast, our proposed approach achieved a higher caliber of results, characterized by sharp edges and enhanced visual quality, thereby indicating its superiority in maintaining image fidelity while effectively adjusting details.

Besides, we performed quantitative analysis to validate the proposed and prior approaches. The PSNR and SSIM results are shown in Figs. 9 and 10, respectively. Six images are taken from Set12 dataset and the noise level is set to 25. It is evident from the measured PSNR values that EPLL^46^ results have noise and that is the underline reason of low PSNR score of this model. Besides, AVMF^47^ and BM3D^5^ approaches attained almost same PSNR scores. Likewise, DIBS^48^ and FFDNet^14^ models’ PSNR scores are close to each others. Yet, on one images the PSNR score of AVMF is higher than proposed model but our approach demonstrates superior denoising performance for all other images.

**Figure 11:**
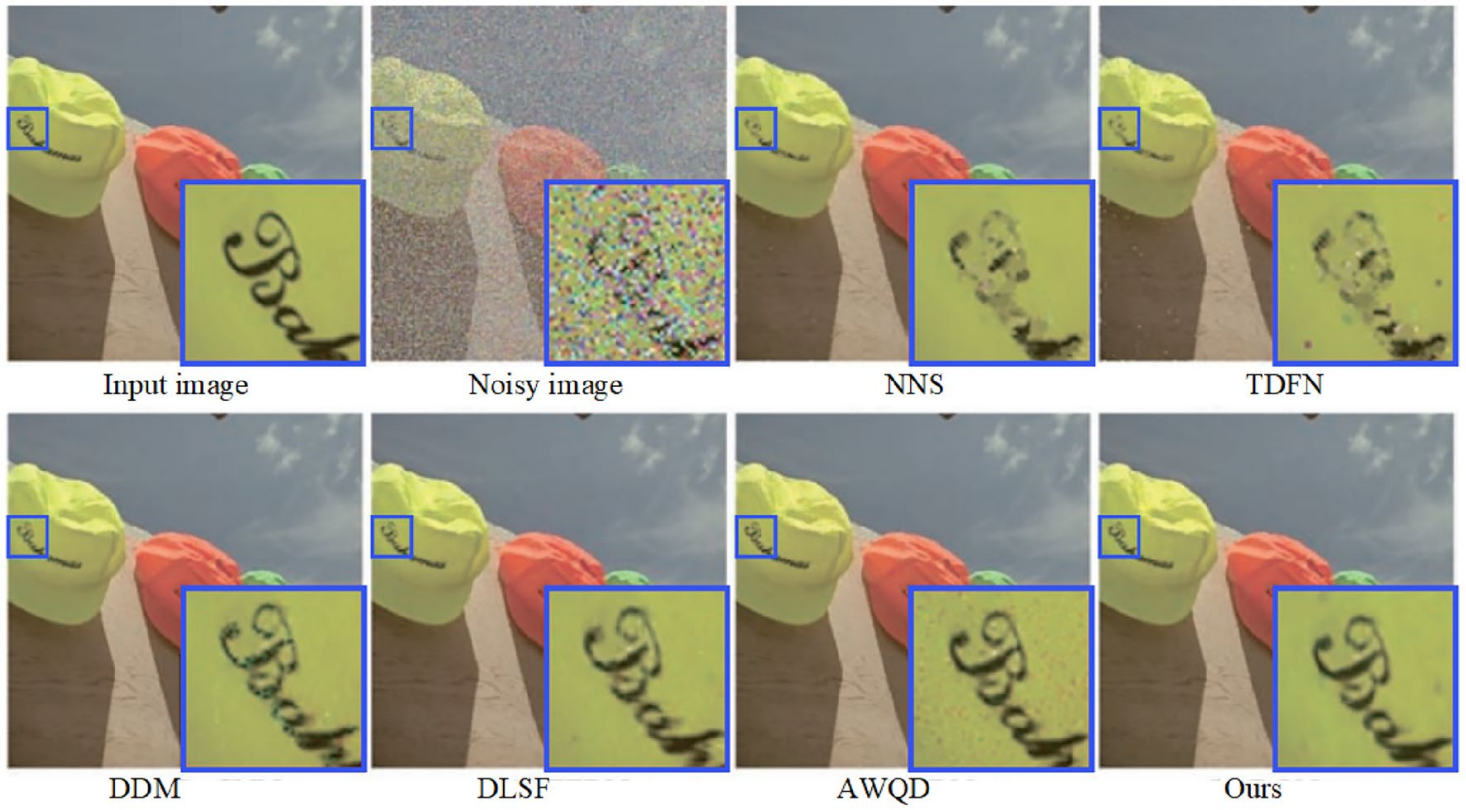
Comparative visual analysis of prior models versus our model on Kodak24 dataset with noise level $\sigma =50$.

In addition to qualitative assessments, a rigorous quantitative analysis is conducted to validate the efficacy of the proposed denoising method against established approaches. The results, encapsulated in terms of PSNR and SSIM, are depicted in Figs. 9 and  10, respectively. For this analysis, a sample of six images from the Set12 dataset is utilized, with the noise level uniformly set at 25. Upon examination of the PSNR values, it becomes apparent that the EPLL^46^ method suffers from residual noise artifacts, which is the fundamental cause for its relatively low PSNR scores. Conversely, AVMF^47^ and BM3D^5^ exhibit comparable PSNR outcomes, indicating a similar level of noise suppression. In parallel, the PSNR scores of the DIBS^48^ and FFDNet^14^ are also closely matched, suggesting that these models perform comparably under the test conditions. However, it is noteworthy that while the AVMF method achieved a higher PSNR score than the proposed model in one instance, our approach consistently demonstrates superior denoising performance across the remainder of the image set.

**Figure 12:**
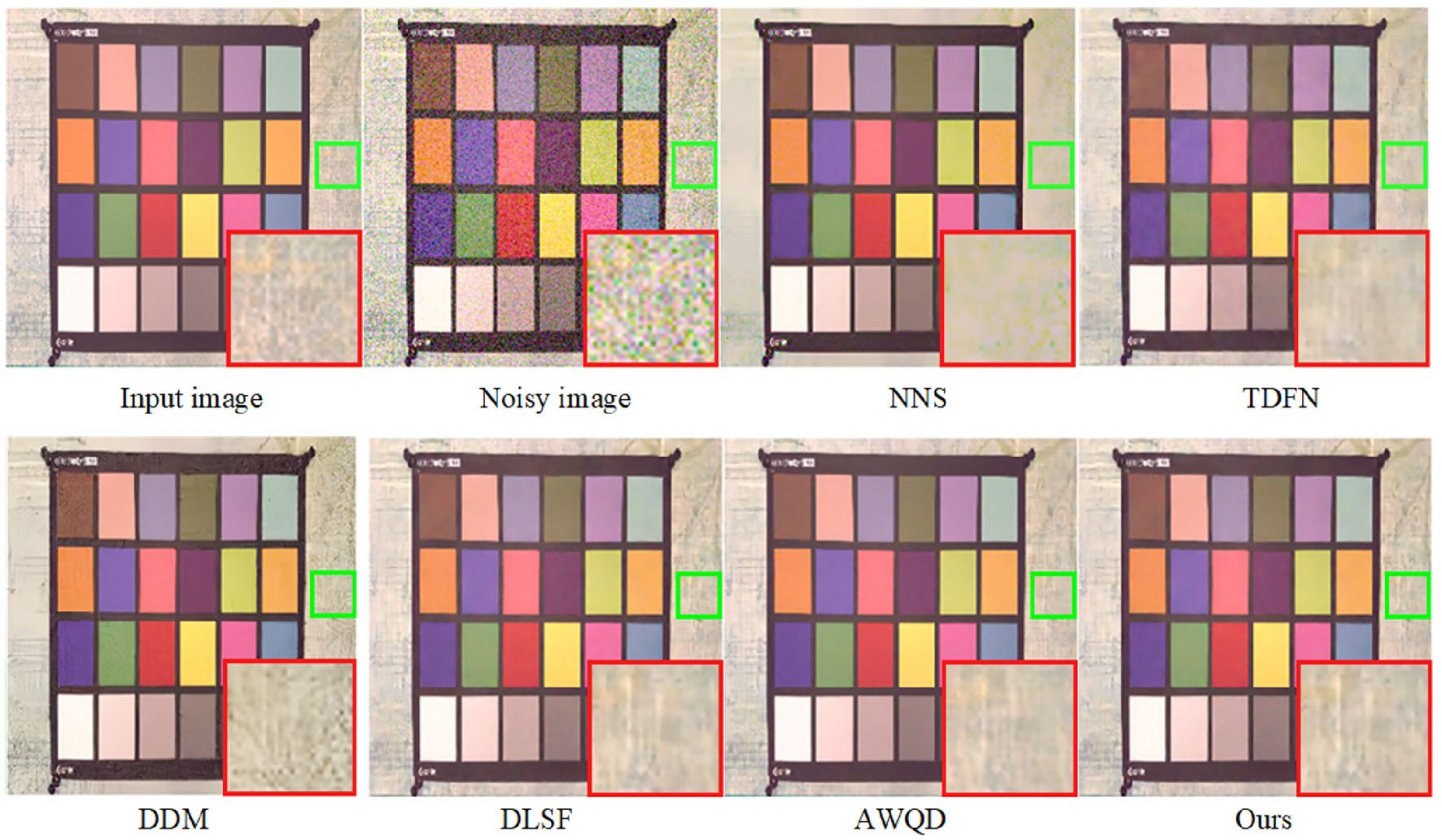
Color image denoising of prior models versus our model on SIDD dataset with noise level $\sigma =50$.

**Figure 13:**
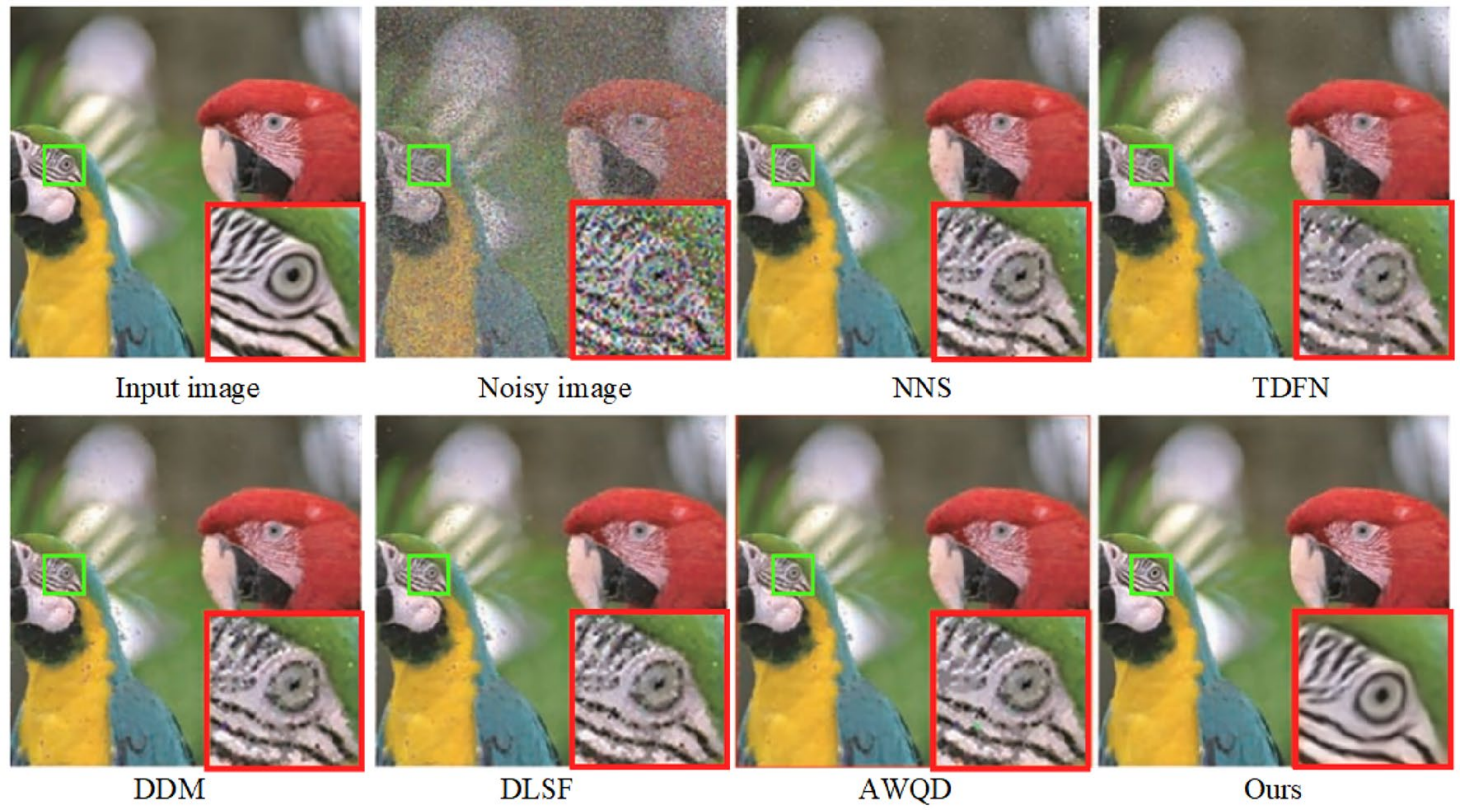
A comparison of the denoising effectiveness of traditional methods and the proposed method with noise level $\sigma =50$.

**Figure 14:**
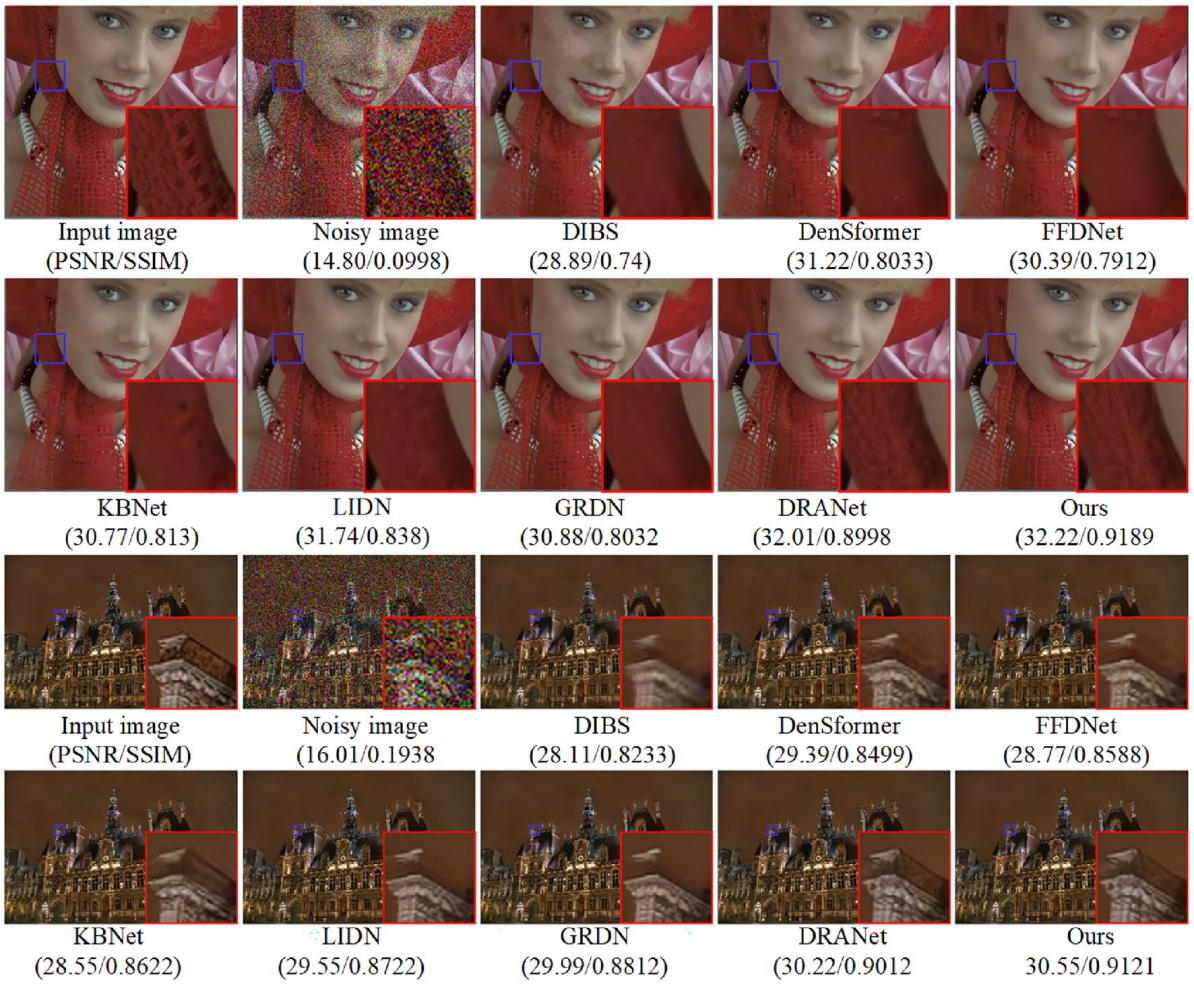
A visual comparison of denoising results that demonstrates the performance of proposed methods versus previous approaches in real-world scenarios with noise level $\sigma =15, 30, 50$.

#### Color image denoising

In addressing real noise scenarios, our study utilized the SIDD^49^. The SIDD encompasses a comprehensive training set and a separate testing suite, with the former containing subsets of varied scales: small (160 noisy-clean image pairs), medium (320 pairs), and large (24,000 pairs). The model retraining is conducted using the small-scale subset of the SIDD training collection, employing data augmentation techniques such as horizontal and vertical flipping to enhance the robustness of the findings. Given that the SIDD test suite does not include corresponding clean images, it is leveraged solely for the purpose of assessing denoising performance. This suite comprises 40 images, from which we extracted three distinct samples representing varying levels of scene brightness to conduct an in-depth comparative study of single-image denoising metrics. These images are resized prior to testing to ensure a clear distinction from those used during the training phase. The denoising outcomes are depicted in Figs. 11 and 12, revealed that while NNS^50^ can mitigate a portion of the real noise, it inadvertently introduces blurriness to the images. The TDFN^51^ approach suppressed a considerable amount of noise but the edges are not very clear. The DDM^52^ technique, while effective at removing substantial noise, also results in a degree of image blur. Conversely, techniques like DLSF^53^ and AWQD^54^ exhibit superior noise elimination proficiency, resulting in images of aesthetically satisfying sharpness. However, the method proposed in this study achieves enhanced restoration of textural and structural details in comparison to the aforementioned models.

A closer inspection of the denoising results within the ROI for each scene in Figs. 11 and 12 unveiled that proposed method consistently retains minimal residual noise. This nuanced analysis underscored the advanced denoising capabilities of the proposed algorithm, which holds promise for setting a new standard in image restoration practices amidst varied lighting conditions.

**Figure 15:**
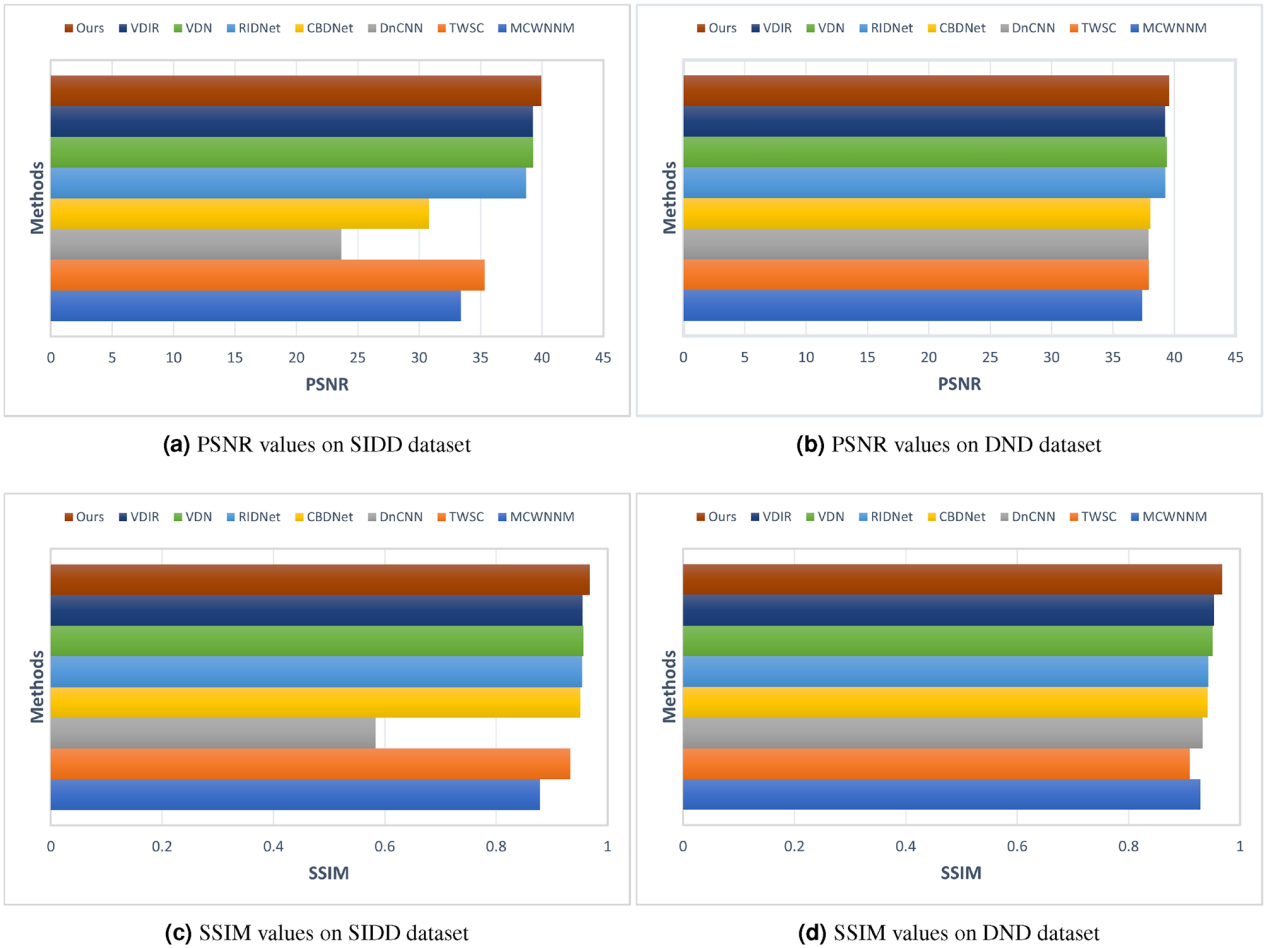
Evaluation of denoising performance in terms of average PSNR and SSIM across SIDD and DND datasets with noise level $\sigma =50$.

Moreover, Fig. 13 presents the results of the proposed method compared to traditional denoising methods such as NNS, TDFN, DDM, DLSF, and AWQD when dealing with random impulse noise in a parrot image with a noise density of 50. This image is taken from the Kodak24 dataset. From Fig. 13, it can be observed that the results obtained by prior denoising methods still contain some noticeable noise, and the details are not well-preserved. In contrast, our model effectively removed noise while preserved the edge and texture details of the image. Hence, our model demonstrated superior performance compared to other algorithms. It generated denoised images that retained a much closer resemblance to the original, particularly under high noise levels. The other prior algorithms included in the comparison exhibited various shortcomings in their denoised outputs. These issues manifested as artifacts, distortions, and even residual noise remaining in the images.

Additionally, the performance of both proposed and existing models is evaluated within a real-world context. This assessment is illustrated through visual results depicted in Fig. 14, which sources its images from two datasets known for their real-world applicability, namely Kodak24 and Urban100. The experiment is conducted with both sets of images subjected to a noise level of $\sigma =50$. Models such as DIBS^48^, DenSformer^55^, FFDNet^14^, KBNet^56^, and GRDN^22^ demonstrated improved outcomes; however, the resultant smooth effect tended to obscure the textures. Conversely, LIDN^57^ and DRANet^58^ managed to enhance the image quality satisfactorily, preserving texture to a certain extent while effectively reducing noise. In contrast, our model distinguishes itself by efficiently removing noise while also preserving the sharpness of edges and the textures in the images. Furthermore, it exhibited unparalleled performance metrics, notably in PSNR and SSIM, surpassing other algorithms. It succeeded in producing denoised images that closely mirror the original, especially under conditions of high noise levels. The comparison also highlighted the limitations of other algorithms, which included various issues such as artifacts, an overly smoothed appearance, and the presence of residual noise, underscoring the superior efficacy of our model in handling real-world imaging challenges.

To further solidifying efficacy of the model, our proposed method achieved demonstrably superior performance in two additional experiments employing diverse datasets,i.e., SIDD and DND. Focusing on a noise level of $\sigma = 50$ and utilizing PSNR and SSIM as metrics, our method surpassed all other compared network models, as showcased in Fig. 15a. MCWNNM^59^ model achieved a PSNR of 33.4, while the TWSC^60^ showed a notable improvement with a PSNR of 35.33. The DnCNN^12^, although an influential model, lags behind with a PSNR of 23.66, which suggests a potential deficiency in handling the noise levels present within the test datasets. CBDNet^15^ offered a moderate performance boost with a PSNR of 30.78. Noteworthy is the RIDNet^61^ model, which demonstrates a significant leap in denoising capability, achieving a PSNR of 38.71. It is closely followed by the VDN^62^ and VDIR^63^ models, with PSNRs of 39.28 and 39.26, respectively, indicating their effectiveness in noise reduction while maintaining high image quality. Most impressively, our proposed model surpassed all compared models with a leading PSNR of 39.94. Likewise, the outcomes in Fig. 15b shown that the proposed model outperformed the prior models. Besides, our method attained higher SSIM and PSNR values on SIDD and DND datasets as shown in Fig. 15c,d. This substantiates the efficacy of our model in denoising images, underscoring the advancements our approach contributes to the field. The empirical evidence strongly suggests that our method sets a new benchmark for denoising performance, potentially redefining the state-of-the-art standards.

To further validate the restoration performance of the proposed model on color images, we performed quantitative analysis in terms of PSNR and FSIMc. The measured results are shown in Table 1 that presents a quantitative scores of various image denoising methods evaluated on the Kodak24 dataset. The proposed method achieved the highest PSNR values across all four different noise level, with scores of 40.77, 36.75, 35.07, and 31.94, respectively. These results indicate a notable improvement in image quality and noise reduction over other methods. Similarly, the proposed method outperformed in terms of FSIMc, achieved the highest scores of 0.998, 0.996, 0.995, and 0.986, suggested it maintained color and structural integrity to a degree superior to that of the other evaluated methods.

**Table 1:** Quantitative analysis of the proposed and prior approaches on the Kodak24 dataset with noise level $\sigma =15, 30, 50$.

Methods	Year	PSNR				FSIMc			
EPLL^46^	2011	30.96	28.06	24.91	20.12	0.986	0.962	0.923	0.823
AVMF^47^	2017	28.76	26.99	24.35	19.97	0.972	0.956	0.918	0.818
DnCNN^12^	2017	37.58	35.62	34.12	31.75	0.995	0.992	0.988	0.979
TWSC^60^	2018	29.93	24.86	20.44	15.75	0.98	0.947	0.879	0.741
FFDNet^14^	2018	38.93	36.61	34.21	31.17	0.997	0.994	0.989	0.978
AWQD^54^	2019	34.1	31.51	29.32	26.41	0.991	0.985	0.973	0.95
DLSF^53^	2020	33.59	30.96	28.85	25.68	0.992	0.981	0.969	0.933
GCDN^64^	2020	28.93	27.78	26.33	22.38	0.973	0.952	0.945	0.852
ADNet^65^	2020	40.52	36.23	34.03	31.73	0.997	0.994	0.991	0.983
BM3D^5^	2022	26.79	25.87	25.09	23.96	0.943	0.937	0.924	0.898
TDFN^51^	2023	29.41	26.88	25.39	23.11	0.976	0.945	0.93	0.883
NNS^50^	2023	30.58	28.1	26.15	23.59	0.98	0.968	0.947	0.897
DDM^52^	2023	33.02	30.22	27.93	24.82	0.989	0.982	0.966	0.924
Ours	–	**40.77**	**36.75**	**35.07**	**31.94**	**0.998**	**0.996**	**0.995**	**0.986**

Significant values are given in bold.

FFDNet and ADNet also showed robust performance with PSNR values exceeding 31 and FSIMc scores above 0.978 in their worst cases, indicating strong denoising capabilities. These methods and our approach stand out as significant contributors to the field of image denoising. Conversely, older methods like EPLL and BM3D showed lower PSNR and FSIMc scores, which could be indicative of the rapid evolution and improvement in denoising techniques over the last decade. From this data, it is clear that the proposed method represents a substantial advancement in denoising technology, setting a new benchmark for both PSNR and FSIMc metrics on the Kodak24 dataset. This suggests that the method could be highly effective in practical applications where maintaining image quality is critical.

Furthermore, we performed another comprehensive quantitative analysis of various image denoising methods on the PolyU dataset, which is also evaluated in terms of PSNR and FSIMc as shown in Table 2. The proposed method achieved the highest PSNR values at 52.09, 47.01, 46.48, and 42.12, and dominated in FSIMc scores with 0.999, 0.998, 0.998, and 0.994 across the four noise level. These results indicated that the proposed method not only excels in reducing noise but also maintained image quality effectively. Other noteworthy methods include ADNet and FFDNet. Specifically, ADNet demonstrated a significant PSNR value of 52.03 and the highest FSIMc score of 0.999 in one category, indicating its strong denoising capability. FFDNet also showed remarkable results, especially with the highest PSNR score of 47.17 in one category. Comparatively, earlier techniques like EPLL and AVMF registered lower scores in both PSNR and FSIMc, reflecting the advancements in denoising technology over the past decade.

Overall, the proposed method exhibited superior performance, not only in noise reduction but also in preserving image fidelity, as reflected in the high FSIMc scores.

**Table 2:** Quantitative analysis of the proposed and prior approaches on the PolyU dataset with noise level $\sigma =15, 30, 50$.

Methods	Year	PSNR				FSIMc			
EPLL^46^	2011	40.32	33.08	26.84	19.95	0.995	0.973	0.901	0.718
AVMF^47^	2017	39.34	32.58	26.58	18.85	0.992	0.966	0.893	0.713
DnCNN^12^	2017	43.02	41.69	42.22	40.55	0.996	0.994	0.99	0.989
FFDNet^14^	2018	46.8	**47.17**	44.79	40.65	0.998	0.998	0.997	0.991
TWSC^60^	2018	35.4	26.65	20.92	15.4	0.989	0.929	0.802	0.588
AWQD^54^	2019	45.81	42.29	39.27	33.04	**0.998**	0.997	0.992	0.964
ADNet^65^	2020	52.03	45.37	42.35	40.16	**0.999**	0.997	0.996	0.993
GCDN^64^	2020	42.21	38.42	32.73	23.92	0.996	0.992	0.964	0.812
DLSF^53^	2020	40.7	37.86	35.27	30.04	0.996	0.992	0.983	0.937
BM3D^5^	2022	39.64	37.57	35.94	33.62	0.995	0.992	0.987	0.977
DDM^52^	2023	38.7	35.93	33.48	28.78	0.993	0.987	0.975	0.921
TDFN^51^	2023	30.78	28.72	27.42	25.32	0.963	0.943	0.924	0.868
NNS^50^	2023	32.31	29.98	28.27	25.7	0.974	0.957	0.936	0.884
Ours	–	**52.09**	47.01	**46.48**	**42.12**	**0.999**	**0.998**	**0.998**	**0.994**

Significant values are given in bold.

### Computational complexity analysis

In our study, we conducted a thorough comparison of various deep learning denoising algorithms against our proposed model to evaluate computational efficiency. We standardized the network input to an image size of $256\times 256$ across three channels for a fair comparison. The parameters and computational complexities for each considered network are meticulously detailed in Table 3, which also includes execution times under a uniform testing environment for images of identical size. Our investigation revealed that our model significantly outperforms established methods such as ADNet, S2S-LSD, FFDNet, RIDNet, and DnCNN in terms of denoising efficiency, demonstrating a noteworthy reduction in execution time. This efficiency gain is largely due to our model’s optimized network structure, which achieves a balance between depth and computational demand, unlike some prior algorithms whose deeper structures lead to increased parameters and complexity. Moreover, we introduced additional contemporary methods to the comparison, further highlighting our model’s advanced capabilities and efficiency.

**Table 3:** Extended comparative analysis of computational complexity.

Method	Running time/s (per image)	Time to train the model	FLOPs/10⌃(9)	Parameter/10⌃(6)
ADNet^65^	0.86	–	1.36	0.52
S2S-LSD^66^	0.91	–	1.27	0.48
FFDNet^14^	0.65	18 hours	1.30	0.50
DnCNN^12^	0.98	–	1.46	0.55
RIDNet^67^	0.75	20 hours	1.40	0.60
Ours	0.21	14 h	1.11	0.42

This extended analysis not only confirms the superior denoising performance of our method but also showcases its remarkable efficiency and practicality for real-world applications. By integrating a streamlined architecture, our model demonstrates an exceptional capability to deliver high-quality denoising results.

## Limitations

Transforming image denoising models that are initially designed for grayscale images to efficiently process color images is a complex endeavor. Such models, especially those employing advanced techniques like Progressive Residual and Convolutional Attention Feature Fusion, often struggle to accurately capture and maintain the essential connections between different color channels. This accuracy is vital for ensuring that the colors in the denoised images remain true to the original. The intricacy of this challenge increases significantly when these models are faced with unfamiliar or sophisticated noise patterns that deviate from standard training scenarios. Often, the datasets used to train these models do not encompass the full spectrum of noise found in real-world settings, which hampers the model’s ability to effectively apply its denoising capabilities across diverse types of noise. To overcome these hurdles, it is imperative to direct future research efforts towards the creation of more advanced learning mechanisms. These mechanisms should be adept at discerning the subtle dependencies between color channels and enhancing the model’s resilience against a broad array of noise distributions. Improving the model’s architecture and pioneering novel training methodologies are crucial steps in this direction. By incorporating a wider array of noise characteristics into the training process, these advancements will equip the model with the versatility needed to tackle the unpredictable and varied nature of noise in real-world images. This approach not only promises to elevate the performance of image denoising models on color images but also aims to bridge the gap between theoretical models and practical applications, ensuring that denoising technologies can meet the demands of real-world challenges with greater efficacy.

## Concluding remarks

In this article, we critically examined the prevalent issues associated with current deep learning approaches to image denoising, i.e., the excessive depth and large parameter sets of networks, which consequentially impede denoising velocity. To navigate these challenges, we integrated the merits of dense block architectures and residual learning frameworks, coupled with a sequential fusion strategy. Consequently, we introduced an innovative sequential residual fusion dense network tailored for mitigating Gaussian noise and real-world noise. Our proposed methodology commenced with the deployment of dense blocks, meticulously engineered to map the distribution of noise within the images. This initial phase significantly streamlines the network’s parameters, simultaneously facilitating an exhaustive extraction of local image attributes. Subsequently, the network employs a methodical approach, progressively amalgamating superficial convolutional features with their more profound counterparts. This step-by-step integration gives rise to a robust residual fusion infrastructure, proficient in harvesting a comprehensive array of global features pertinent to the identified noise. This procedure reaches its zenith with the amalgamation of the resultant feature maps emanating from each dense block. Besides, a tripartite attention mechanism called CAFFM is employed to compute relative attention weights that reflect the interconnectedness of three distinct dimensions. These weights are then validly applied to a duo of feature planes targeted for fusion. This non-linear methodology for merging features is adept at identifying the interactions among multiple feature planes, thereby substantially augmenting the effectiveness of the fusion procedure. These are then adeptly channeled towards the reconstruction layer, which is responsible for synthesizing the final denoised image output. This sophisticated architecture ensures a fusion of both depth and precision, culminating in an efficient and effective denoising process. Empirical studies in environments with Gaussian white noise and natural noise showed a significant performance improvement. This is evidenced by higher mean values in PSNR, SSIM, and FSIMc, outperformed more than 20 existing methods across six different datasets.

## Data Availability

The datasets used and/or analysed during the current study available from the corresponding author on reasonable request.
