# Author Correction: An efficient lightweight network for image denoising using progressive residual and convolutional attention feature fusion

**DOI:** 10.1038/s41598-024-66739-x

**Published:** 2024-07-09

**Authors:** Wang Tiantian, Zhihua Hu, Yurong Guan

**Affiliations:** 1 School of Computer and Software Engineering, Sias University, Zhengzhou, 451150 Henan China; 2 School of Computer, Huanggang Normal University, Huanggang, 438000 Hubei China

Correction to: *Scientific Reports* 10.1038/s41598-024-60139-x, published online 25 April 2024

The original version of this Article contained an error in Affiliation 1, where the university was incorrectly stated as ‘Xias University’.

The correct affiliation is listed below.

^1^School of Computer and Software Engineering, Sias University, Zhengzhou, 451150, Henan, China.

The original Article has been corrected.

